# pH-Driven Polymorphism of Insulin Amyloid-Like Fibrils

**DOI:** 10.1371/journal.pone.0136602

**Published:** 2015-08-27

**Authors:** Tomas Sneideris, Domantas Darguzis, Akvile Botyriute, Martynas Grigaliunas, Roland Winter, Vytautas Smirnovas

**Affiliations:** 1 Department of Biothermodynamics and Drug Design, Vilnius University Institute of Biotechnology, Vilnius, Lithuania; 2 Physical Chemistry I–Biophysical Chemistry, Faculty of Chemistry and Chemical Biology, TU Dortmund University, Dortmund, Germany; Rocky Mountain Laboratories, NIAID, NIH, UNITED STATES

## Abstract

Prions are infective proteins, which can self-assemble into different strain conformations, leading to different disease phenotypes. An increasing number of studies suggest that prion-like self-propagation may be a common feature of amyloid-like structures. Thus it is important to unravel every possible factor leading to the formation of different amyloid strains. Here we report on the formation of two types of insulin amyloid-like fibrils with distinct infrared spectroscopic features grown under slightly different pH conditions. Similar to prion strains, both insulin fibril types are able to self-propagate their conformational template under conditions, favoring spontaneous formation of different type fibrils. The low-pH-induced insulin amyloid strain is structurally very similar to previously reported strains formed either in the presence of 20% ethanol, or by modification of the amino acid sequence of insulin. A deeper analysis of literature data in the context of our current findings suggests a shift of the monomer-dimer equilibrium of insulin as a possible factor controlling the formation of different strains.

## Introduction

Amyloid-like structures are associated with a number of pathological conditions including neurodegenerative diseases, such as Alzheimer’s and Parkinson’s, and infectious prion diseases, also a number of nonneuropathic systemic amyloidoses, and even type II diabetes [1]. In some cases amyloid-like folds can play a positive role as well: they have a structural function in spider silk and biofilm formation in bacteria, and a regulatory function in fungi or hormone storage in humans [2]. Experiments *in vitro* revealed even more amyloid-forming proteins and peptides, including proteins with no link to *in vivo* amyloids, such as polyaminoacids (e.g., polylysine, polythreonine and polyglutamic acid) [3], and short oligopeptides [4–6]. Finally, even an amyloid-like self-assembly of phenylalanine was recently reported [7]. All these findings support the idea that amyloid-like folds may be a generic property of all polypeptides, while the propensity of fibril formation would depend on the sequence of the polypeptide and on the environmental conditions (i.e., temperature, pressure, solution milieu, interaction with lipid interfaces, pH) [1].

Prions stand out among other amyloid-forming proteins as the only proteinaceous infectious pathogens [8]. Identical amino acid sequences of prion protein can adopt distinct pathogenic conformations, referred to as prion strains [9,10]. Different strains lead to distinct incubation periods and patterns of neuropathology in prion diseases [10]. Similar conformational variations were detected in other amyloid-forming proteins both *in vitro* [11–22] and i*n vivo* [23–25]. With growing evidence of the involvement of prion-like mechanisms in the progression of other amyloid-related diseases [23–33], it is indispensable to understand all the factors determining formation of different amyloid strains.

The new variant Creutzfeldt-Jakob disease (vCJD) is thought to be caused by a bovine spongiform encephalopathy (BSE) strain [34]. In this case, the determining factor for the formation of distinct prion strains is cross-species infection. Similar to prions, formation of distinct amyloid strains for two slightly different insulin forms was recently reported [19,35]. When protein sequences are identical, the environment plays the key role in straining of amyloid-like fibrils. The presence of co-solvents [11,14,15,20], different temperatures [36–38], different concentrations of denaturants [38,39] and salts [21], or different ways of agitation [12,40] may lead to distinct amyloid fibril strains. Here we report on the formation of distinct insulin amyloid strains at slightly different pH values.

As diagnostic tool, Fourier-transform infrared (FTIR) spectroscopy has been used, which has proven to be an important method for the characterization of secondary structural changes of prion and amyloid strains [11,19,41], supplemented by atomic force microscopy (AFM) measurements of the topology of amyloid fibrils and thioflavin T (ThT) fluorescence for recording the fibrillation kinetics.

## Results and Discussion

**Fig 1:**
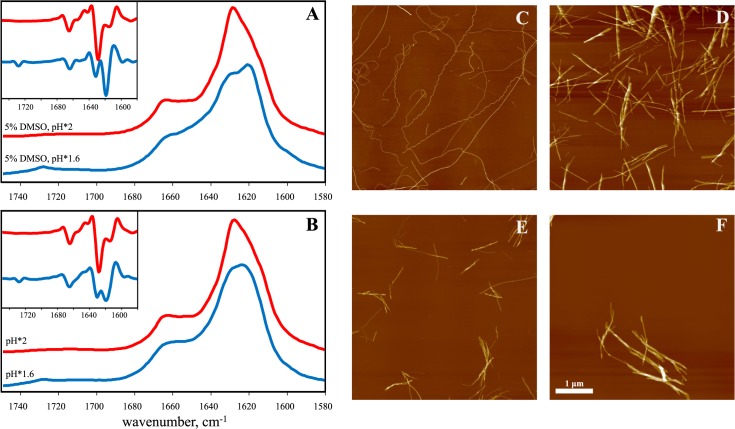
Polymorphism of insulin amyloid-like fibrils formed at different pH* values. FTIR absorption spectra (second derivative spectra in the inset) of fibrils grown in the presence (A), and absence (B) of 5% DMSO (spectra were repeated using different FTIR instruments in different labs, see S1 Fig). AFM images of fibrils prepared in the presence of DMSO at pH*1.6 (C), and pH*2 (D) or in absence of DMSO at pH*1.6 (E), and pH*2 (F). Fibril height measurements are shown in S2 Fig.

In our recent work on potential inhibitors of insulin amyloid-like fibrillation, we followed the aggregation of insulin at pH 2 in the presence of 5% residual dimethylsulfoxide (DMSO) [42]. To test if the presence of a small amount of DMSO affects the fibrillation process, we compared the FTIR spectra of insulin amyloid-like fibrils spontaneously formed in D_2_O in the presence (Fig 1A) and absence (Fig 1B) of 5% DMSO. To reveal possible changes upon using D_2_O instead of H_2_O, as required for the better quality FTIR measurements, and for looking into subtle pH changes on the fibrillation propensity of insulin, fibrils were prepared in heavy water samples at two pH* values (where pH* is the pH-meter readout uncorrected for isotopic effects, see Methods section), pH*1.6 to mimic similar concentrations of H^+^ and D^+^, and pH*2 to reach the same ionization state of the protein in the two solvents. The FTIR spectra look similar in the presence and absence of DMSO, but a rather small difference in pH* leads to significant differences in amide I’ band contours (Fig 1A and 1B). Spectra of fibrils prepared at pH*2 exhibit maxima in the amide I’ region at ~1628 cm^-1^ (with the main minimum of the second derivative at 1628 cm^-1^ and a weaker one at 1615 cm^-1^), while spectra of fibrils grown at pH*1.6 exhibit maxima in the amide I’ region at ~1621–22 cm^-1^ (with the main minimum of the second derivative at 1619 cm^-1^ and a weaker one at 1631 cm^-1^), pointing toward predominantly beta-sheet structures but with a significantly different hydrogen-bonding patterns. A small band outside of the amide I’ region at ~1728 cm^-1^ is present only in the spectra of fibrils grown at pH*1.6 and can be attributed to deuterated carboxyl groups [19]. Very similar spectral characteristics were recently described as a hallmark of two different insulin amyloid strains [19,35].

Fibrils grown at pH*1.6 in the presence of 5% DMSO are usually 2–4 nm in diameter and exhibit both a curved and straight morphology (Fig 1C), while fibrils grown at pH*2 both in the presence (Fig 1D) and absence (Fig 1E) of DMSO are thicker (4–16 nm) and usually straight. The structure of the fibrils at pH*1.6 in the absence of DMSO (Fig 1E) looks similar to the case at pH*2, suggesting no clear morphological differences between strains.

**Fig 2:**
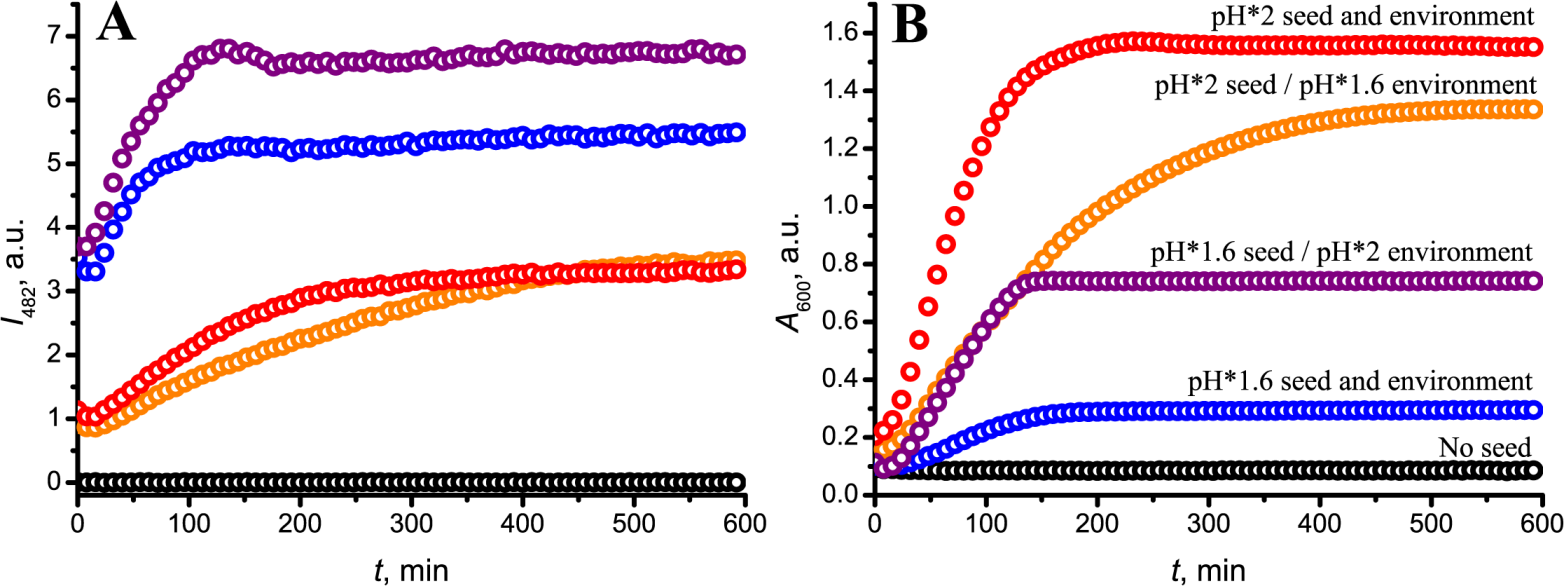
Kinetics of seed-induced aggregation of insulin; followed by ThT fluorescence intensity (A) as maker of fibril formation, and light absorbance at 600 nm (B). Measurements were repeated using 3 batch preparations showing similar results.

Both types of fibril seeds induce aggregation of insulin at either pH* and 37°C (Fig 2). Seeds grown at pH*1.6 fibrillate insulin at similar rates under both pH* conditions and faster than seeds grown at pH*2. The latter seeds elongate faster at solution conditions of the same pH*. As clearly visible, the fluorescence intensity of Thioflavin T (ThT), which marks formation of fibrillar amyloid states, is seed-dependent: pH*1.6-seed-induced aggregates result in an about double ThT intensity when compared to pH*2-seed-induced aggregates (Fig 2A). The light absorbance data at 600 nm—as measure of formation of larger insulin aggregates due to light scattering—show the reverse effect (Fig 2B). The pH*2-type fibrils induce aggregates which strongly absorb visible light (600 nm), the absorbance being ~25% lower in the case of seeding in the pH*1.6 environment. pH*1.6-type fibrils induce weakly absorbing aggregates (about 5 times lower than pH*2-type fibrils); however, the absorbance is strongly increased in the pH*2 solution.

**Fig 3:**
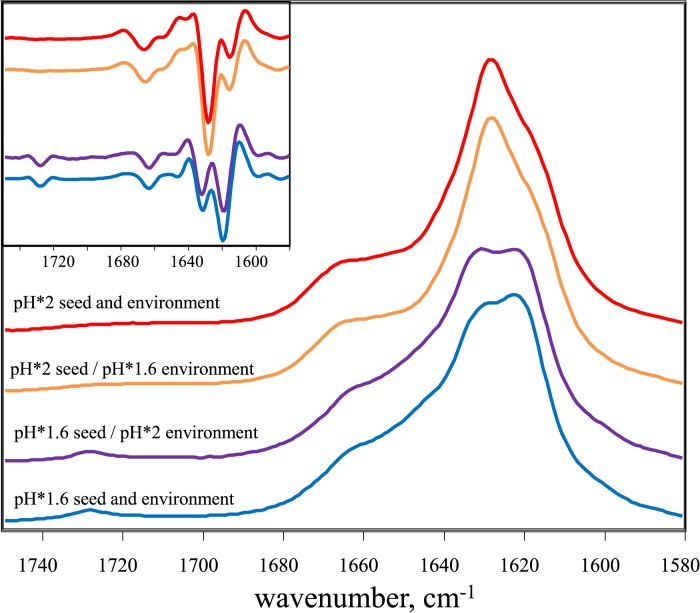
Infrared spectral features determined by the seeding template. Absorption and second derivative (inset) FTIR spectra.

The FTIR spectra of the seeded fibrils clearly demonstrate the superiority of the seed template versus the pH*-environment in controlling the fibrillar structure (Fig 3). The spectra of pH*2-seed-induced aggregates grown at pH*2 and pH*1.6 look identical. In case of the pH*1.6-seeded aggregates, the spectral signature is similar for both solution conditions; however, in pH*2, the intensity of the band at 1631 cm^-1^ is increased. These data confirm the ability of both types of insulin fibrils to self-propagate their conformational template in spite of unfavorable environmental factors (here different pH conditions), suggesting the existence of two different insulin amyloid strains.

**Fig 4:**
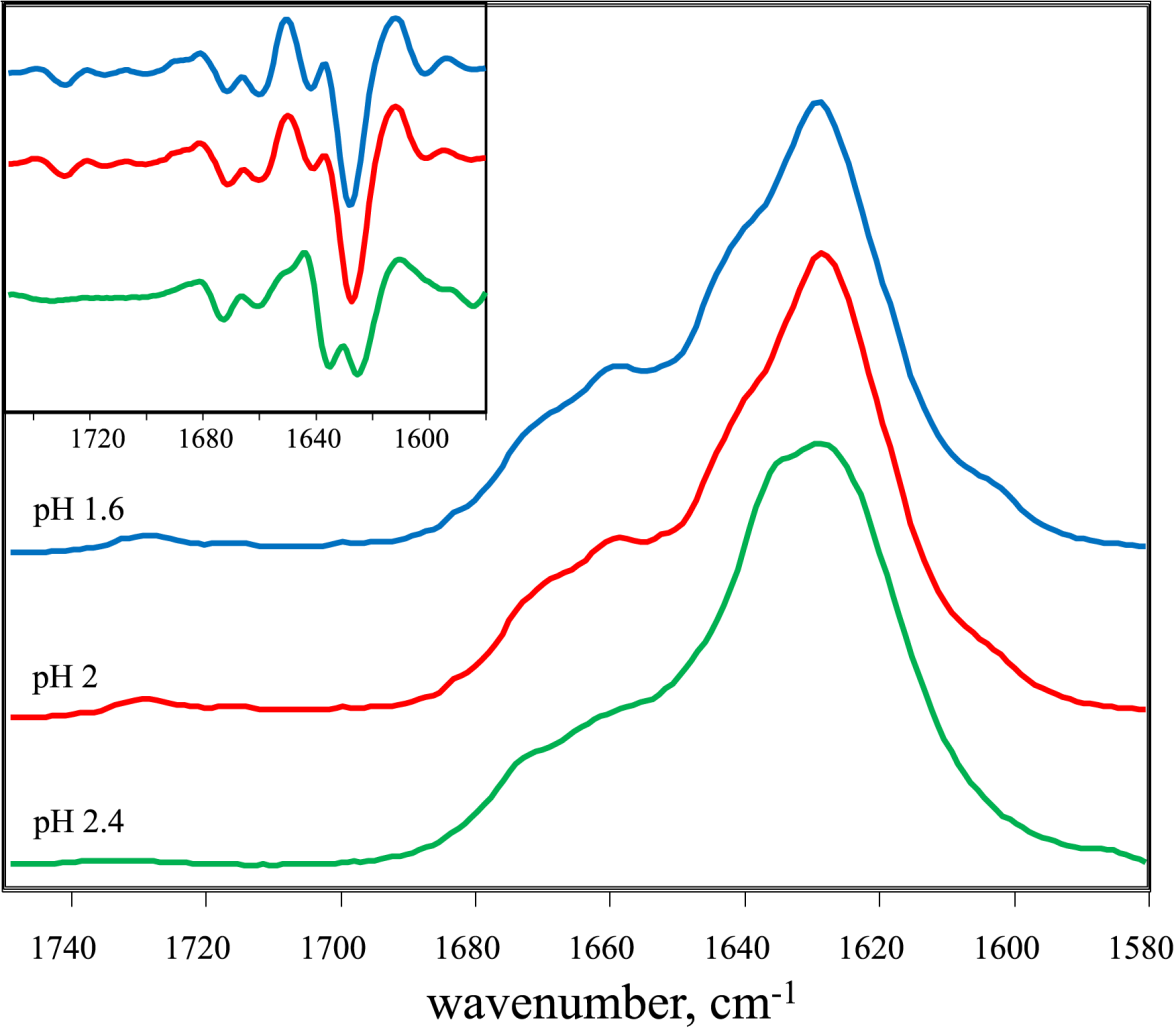
Infrared spectra of insulin amyloid-like fibrils formed in normal water (H_2_O). Absorption and second derivative (inset) FTIR spectra.

Surprisingly, the FITR spectra of insulin amyloid-like fibrils spontaneously formed in H_2_O at pH 1.6 and pH 2 look almost identical (Fig 4). Both spectra exhibit maxima in the amide I/I’ region at ~1628 cm^-1^ (with the main minimum of the second derivative at 1628 cm^-1^ and a weaker one at 1641 cm^-1^), and a small band outside of the amide I/I’ region at ~1730 cm^-1^. A different spectrum was obtained using fibrils spontaneously formed in H_2_O at a slightly higher pH, at pH 2.4: it also exhibits a maximum in the amide I/I’ region at ~1628 cm^-1^, but the second derivative profile is different–two similar sized bands, at 1625 cm^-1^ and 1636 cm^-1^, respectively. As the amyloid-like fibrils are highly protected from hydrogen/deuterium exchange, most of the amide hydrogens stay unchanged despite resuspension of the aggregates in D_2_O. It reflects in the blue-shift of the spectra compared to insulin fibrils, prepared in D_2_O.

Different types of insulin fibrils were first mentioned more than 60 years ago [43], however no structural or cross-seeding data were presented. In more recent studies, formation of different strains were reported in the presence and absence of 20% ethanol (at pH*1.5–1.8) [11,14,15], and using slightly different insulin forms (bovine insulin (BI) and recombinant Lys^B31^-Arg^B32^ human insulin analog (KR)) at pH*1.9 [19,35]. Spectral characteristics of the latter strains are very similar to our data. The spectrum of the fibrils formed at pH*2 is similar to the spectrum of the BI strain, and the spectrum of the fibrils formed at pH*1.6 reminds us of the one of the KR strain. So the effect of two additional positively charged amino acids on the fibrillar structure is similar to the effect of ΔpH by -0.4 units. The change in net charge of the protein due to such ΔpH is minor, and taking into account that in normal water at pH 1.6 and pH 2 insulin aggregates into the same strain, we may conclude that ionization state of the protein is not the factor inducing formation of different strains. So what is the factor?

A possible answer to that question can be found by analyzing recent studies, which, at first sight, seem to contradict our findings [44–46]. In these works, no differences in the FTIR spectra of insulin fibrils formed at different pH values in the range between 1.3 and 3.1 are reported, however, a marked change of the vibrational circular dichroism (VCD) spectra are seen between pH 2.1 and 2.4, which is explained by a different supramolecular chirality [44,45]. Furthermore, it was shown that the chirality can be converted by incubation of preformed fibrils at different pH, thus excluding the possibility of different strains [46]. The reported FTIR spectra lack a detailed description, however, the shape of the amide I band looks very similar to the amide I’ band of the pH*2 fibrils [44–46]. A closer inspection reveals one major experimental difference, which can affect the mechanism of insulin fibrillation. The concentration of insulin used in the aforementioned studies was 60 mg/mL (compared to 1 mM (~5.8 mg/mL) in our study), which means a strong shift towards a higher oligomeric state of insulin in solution, as even at much lower concentrations insulin tends to oligomerize [47–49]. Hence, the factor which determines the formation of different strains could be due to a shift in the monomer-oligomer equilibrium.

**Fig 5:**
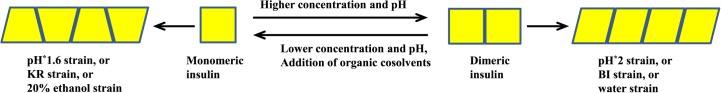
Proposed scheme of insulin amyloid straining.

The spectral features of the insulin amyloid strain formed in the presence of 20% ethanol [11,14,15] are similar to those of the pH*1.6 and KR strain [19,35]. In all three cases the second derivative FTIR spectra in the amide I’ region exhibit strong minima at 1619–1620 cm^-1^, and a weaker one at 1630–31 cm^-1^, plus a small band outside of amide I’ region at 1728–30 cm^-1^. Hence, it should be concluded that the same amyloid strain is present in all three cases. It is known that the presence of 20% ethanol strongly increases the dissociation of insulin dimers [50], leading to predominantly monomeric insulin at moderate concentrations [14,51]. The C-terminal part of the B-chain of insulin is involved in the formation of intramolecular antiparallel β-sheet that binds together native insulin dimers [19]. Thus there is a high probability that two additional charged amino acids would lead to dissociation of dimers in case of KR insulin. There is no data on the monomer-dimer equilibrium of insulin at pH*1.6, but the fact that different strains can be formed not only with increasing pH*, but also with increased concentration of insulin, suggests a shift of the equilibrium to the monomeric state. We may hence hypothesize that the major factor which determines formation of different strains is a shift of the equilibrium between insulin monomers and dimers (oligomers) (Fig 5). If the equilibrium is shifted towards dimers (or higher oligomers), insulin aggregation would result in the pH*2-like strain, and in case that the equilibrium is shifted towards monomers, growth of the pH*1.6-like strain is fostered.

**Fig 6:**
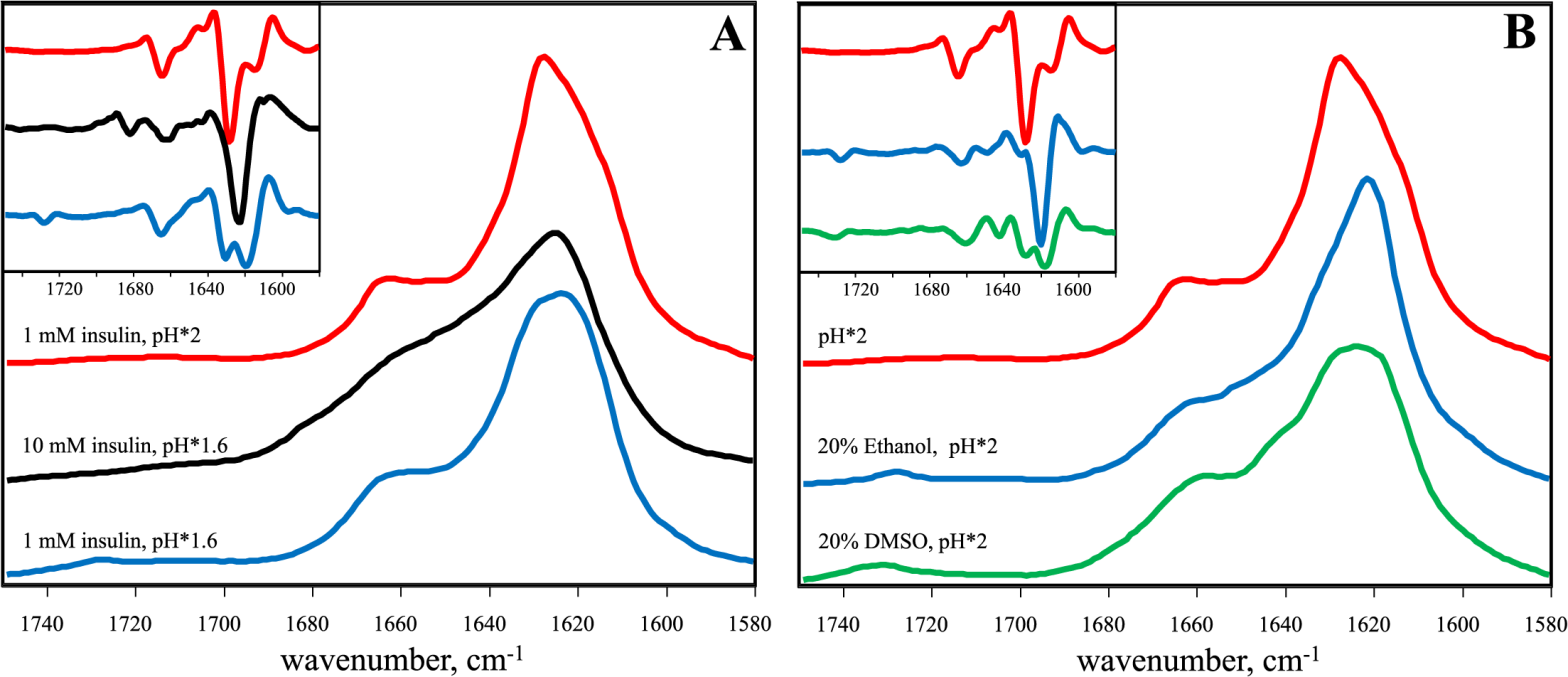
The effect of high insulin concentration (A), and organic cosolvents (B). Absorption and second derivative (inset) FTIR spectra.

To further test the hypothesis we carried out several additional experiments. First we checked if an increased insulin concentration would explain the differences observed between the pH*1.6 and pH*2 data. As seen in Fig 6A, and Table 1, the spectrum of 10 mM insulin aggregates, prepared in the pH*1.6 environment, is slightly different from the other spectra. The blue shift of the amide I’ maximum, when compared to the spectra of the pH*1.6 strain, and the absence of the band around 1728 cm^-1^ suggests that the increased protein concentration leads to formation of different strain. Nevertheless, the spectrum is also different from the pH*2 strain, so this data does not add much to strengthen our hypothesis. It is worth to mention that at high insulin concentration insulin aggregates form gel-like substance (which is not the case at lower insulin concentrations). It may point to the different aggregation mechanism thus explaining difference in FTIR spectra.

**Table 1:** Summary of FTIR band positions of insulin amyloid-like fibrils.

Insulin agregation conditions	Amide I/I’ band (2^nd^ derivative), cm^-1^		Additional bands, cm^-1^
	Beta-sheets	Turns/loops	Carboxyl groups^b^
1 mM insulin, pH*1.6	1619/1631^a^	1665	1728
1 mM insulin, pH*2	1628/1615	1665	absent
1 mM insulin, pH*1.6, 5% DMSO	1619/1631	1664	1728
1 mM insulin, pH*2, 5% DMSO	1628/1615	1665	absent
10 mM insulin, pH*1.6	1623	1662	absent
1 mM insulin, pH*2, 20% ethanol	1620/1631	1663	1728
1 mM insulin, pH*2, 20% DMSO	1619/1629	1661	1731
1 mM insulin, pH 1.6	1628/1641	1672/1661	1729
1 mM insulin, pH 2	1628/1641	1672/1661	1729
1 mM insulin, pH 2.4	1625/1636	1673/1661	absent

^a^All FTIR measurements were repeated at least three times showing similar results.

^b^Band assigned to carboxyl groups according to Surmacz-Chwedoruk et al [19]

We also repeated previously described data on insulin aggregation in the presence of ethanol and examined the effect of higher DMSO concentrations. As seen in Fig 6B, the presence of 20% of both organic cosolvents during insulin aggregation in the pH*2 environment leads to formation of aggregates exhibiting pH*1.6-like IR spectra. This confirms that ethanol and, to a lower extent DMSO shifts the equilibrium towards formation of pH*1.6-like insulin amyloid strains.

**Fig 7:**
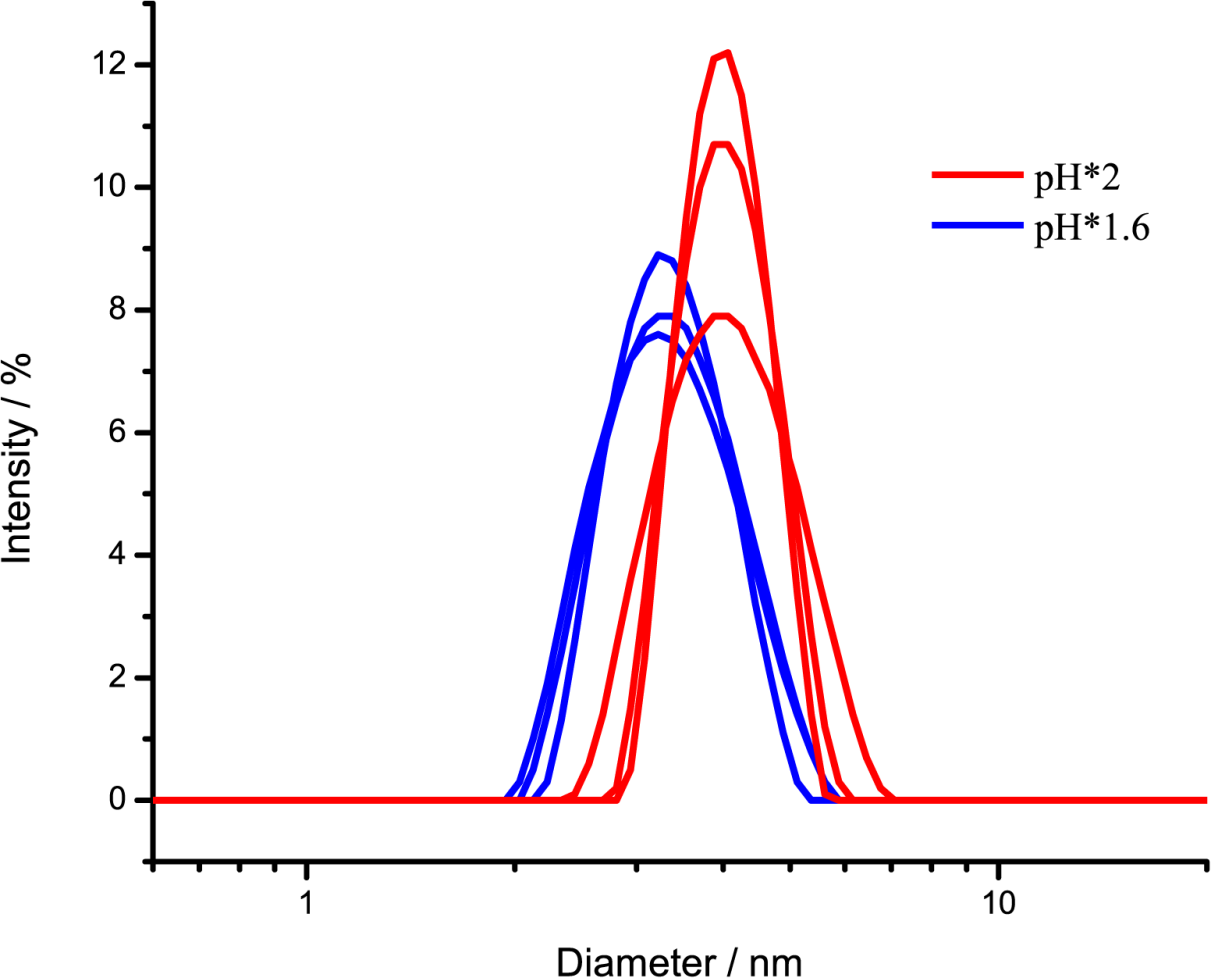
Size distribution of insulin in solution. DLS measurements were repeated using 3 batch preparations with similar results.

Finally, we used dynamic light scattering (DLS) to determine the size distribution of insulin under the various solution conditions. The data reveal that average size of insulin, dissolved in pH*1.6 is lower than in pH*2 (Fig 7). The measured diameter of insulin in pH*1.6 is 3.4±0.7 nm, which is bigger than monomer, but smaller than dimer, while in pH*2, the diameter is 4.0±0.6, which is a little bigger than insulin dimer. Owing to the polydispersity of the sample, the method does not allow the exact estimation of the monomer and oligomer content, however the shift of the equilibrium towards dimeric/oligomeric species at higher pH* is unarguable, hence supporting our hypothesis.

Taken together, our data indicates different factors inducing polymorphism of insulin amyloid-like fibrils. However it seems that all the presented cases can be reduced to the formation of two amyloid strains and possibly explained by the differences in the equilibrium between insulin monomers and dimers (oligomers).

## Materials and Methods

### Preparation of insulin fibrils

Recombinant human insulin was purchased from Sigma Aldrich (91077C). Insulin amyloid-like fibrils were prepared as described previously [52]. Briefly, fresh 1 mM insulin solution (in 100 mM phosphate buffer (PB), at different pH (in H_2_O) and pH* (in D_2_O) values was incubated at 60°C for 24 hours with 300 rpm agitation (using a MHR 23 thermomixer, Ditabis, Germany). The secondary structures and morphological signatures of the aggregates obtained were tested using FTIR spectroscopy and AFM.

Seeds were prepared as described previously [52]. Briefly, 1 mL of fibrils were sonicated for 10 minutes using a Bandelin Sonopuls 3100 ultrasonic homogenizer equipped with a MS73 tip (using 50% of the power, cycles of 30 s/30 s sonication/rest, total energy applied to the sample per cycle, 0.56 kJ). The sample was kept on ice during the sonication procedure. Right after the treatment, one part of the fibrils was mixed with 9 parts of the fresh 1 mM insulin solution in the appropriate buffer and incubated at 37°C for 24 hours without agitation. The secondary structures of the aggregates obtained were tested using FTIR spectroscopy.

### Elongation kinetics

To follow the seeding kinetics, samples were prepared as described above, with addition of 50 μM ThT. Right after the mixing the fresh insulin with seeds, samples were divided into 200 μL aliquots, in 96-well plates. The plates were sealed using clear polyolefin sealing tape. The aggregation kinetics was followed at constant 37°C temperature using a Biotek Synergy H4 plate reader without agitation. ThT fluorescence intensity upon fibril formation was observed using 440 nm excitation and 482 nm emission with simultaneous measurement of absorbance at 600 nm.

### Infrared spectroscopy

To avoid overlapping of protein amide I and water bands, D_2_O is used as solvent in FTIR measurements. At equal concentrations of D^+^ and H^+^, respectively, the pH-meter reading with a glass electrode is 0.4 pH units lower in D_2_O than in H_2_O [53]. However, isotopes affect the pK_a_ of protein ionizable groups, and for solutions of globular proteins the ΔpK_a_ was found to be 0.4 pH units in the acidic range, thus the isotope effect on the glass electrode and the ionization constant cancel each other, so that an identical pH-meter reading (in the acidic range) refers to an identical ionization state of the biopolymer in D_2_O and H_2_O solutions [54]. To prepare samples for the FTIR measurements, insulin fibrils prepared in H_2_O were separated from water by centrifugation (30 min., 15000 *g*), and resuspended in D_2_O, the procedure was repeated three times. All samples were sonicated for 1 minute using a Bandelin Sonopuls 3100 ultrasonic homogenizer equipped with a MS73 tip. The FTIR spectra were recorded using a Nicolet 5700 spectrometer from Thermo Scientific equipped with a liquid-nitrogen-cooled mercury-cadmium-telluride (MCT) detector, and using Bruker Alpha spectrometer equipped with deuterium triglycine sulfate (DTGS) detector. For all measurements, CaF_2_ transmission windows and 0.05 mm Mylar spacers or 0.05 and 0.1 mm Teflon spacers (with Bruker instrument) were used. Spectra were recorded at room temperature. For each spectrum, 256 interferograms of 2 cm^-1^ resolution were co-added. A corresponding buffer spectrum was subtracted from each sample spectrum. All the spectra were baseline-corrected and normalized to the same area of amide I/I’ band (1700–1580 cm^-1^) before further data processing. All data processing was performed using GRAMS software.

### Dynamic light scattering

For DLS experiments, freshly prepared insulin solutions at different buffers were filtered using 0.22 μm syringe filter. The size measurements were performed using Zetasizer μV (Malvern instruments) with low-volume quartz batch cuvette at 60°C.

### Atomic force microscopy

For AFM experiments, 1 mM insulin was diluted 100 times with deionized water, 30 μL of the sample were deposited on freshly cleaved mica and left to adsorb for 1 min, the sample was rinsed with 1 mL of water and dried gently using airflow. AFM images were recorded in the Tapping-in-Air mode at a drive frequency of approximately 300 kHz, using a MultiModee SPM microscope equipped with a NanoScope IIIa controller. PointProbe NCHR aluminium-coated silicon tips from Nanosensors were used as a probe.

## Supporting Information

S1 Fig Example of the repeatability of FTIR spectra. Red and blue spectra were collected using Thermo Nicolet instrument in TU Dortmund University, black and green–using Bruker Alpha instrument in Vilnius University.
(PDF)

S2 Fig Fibril height measurements. (PDF)
