# Supplementary figures and images for: pH-Driven Polymorphism of Insulin Amyloid-Like Fibrils
Source: PLoS One. 2015 Aug 27;10(8):e0136602. doi: 10.1371/journal.pone.0136602 (PMC4551895; doi:10.1371/journal.pone.0136602)

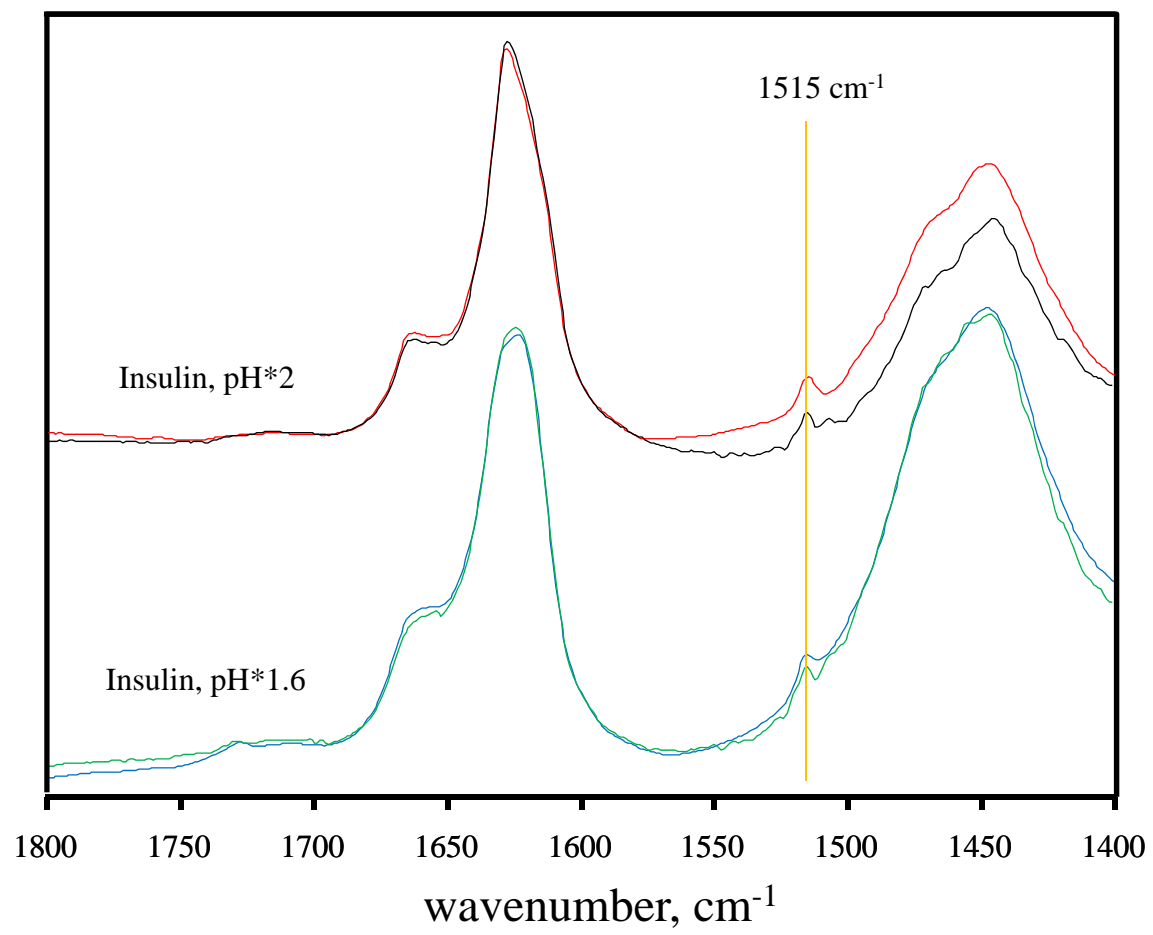

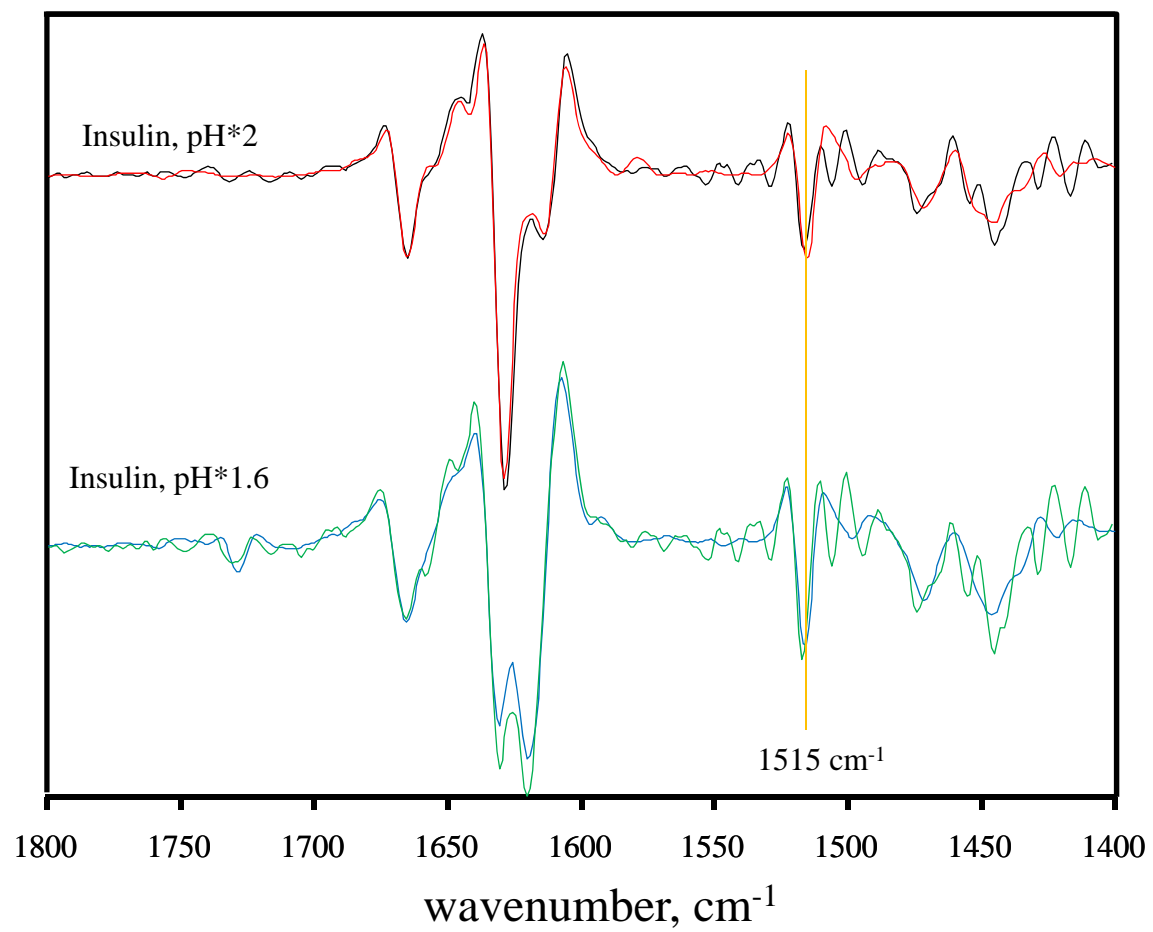

Supplement: S1 Fig — Red and blue spectra were collected using Thermo Nicolet instrument in TU Dortmund University, black and green–using Bruker Alpha instrument in Vilnius University. (PDF) [file pone.0136602.s001.pdf]

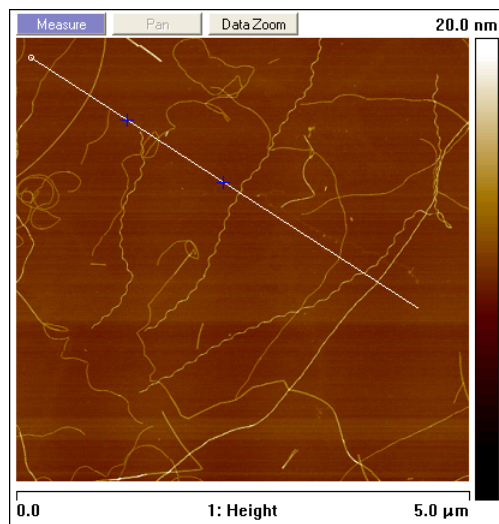

Insulin, 5% DMSO, pH\*1.6

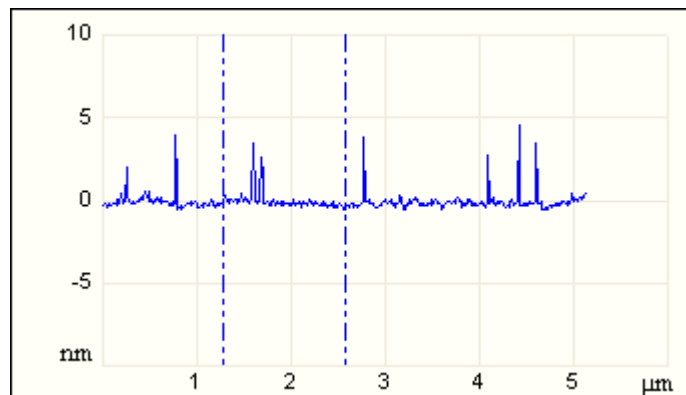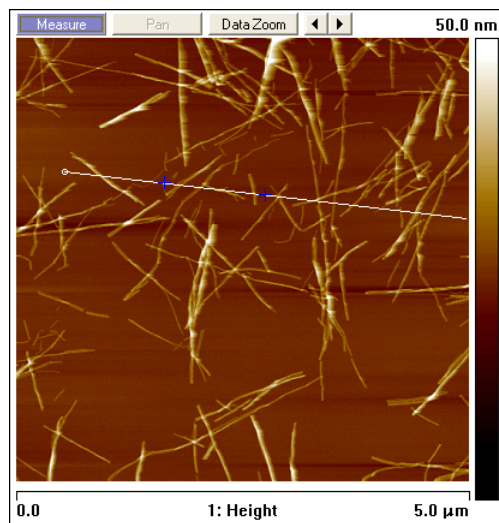

Insulin, 5% DMSO, pH\*2

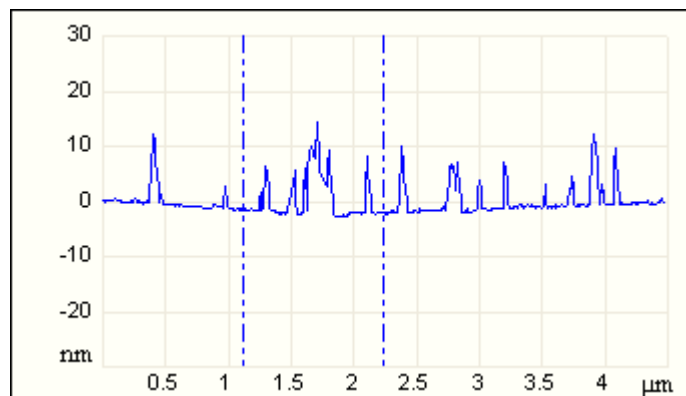

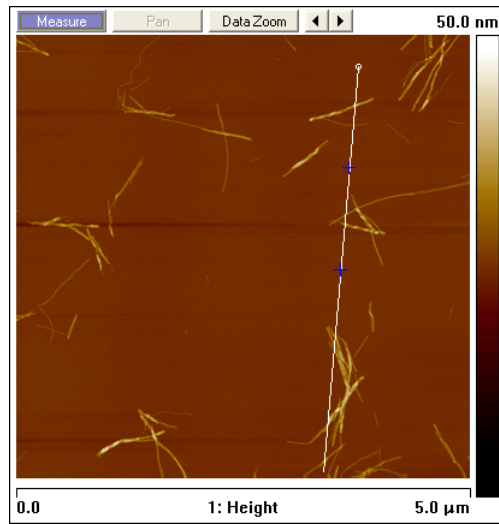

Insulin, pH\*1.6

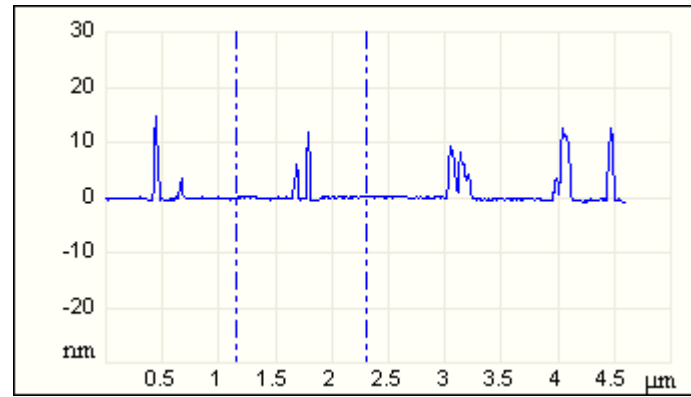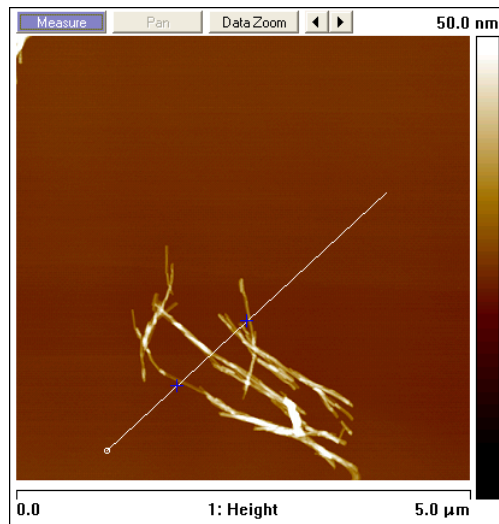

Insulin, pH\*2

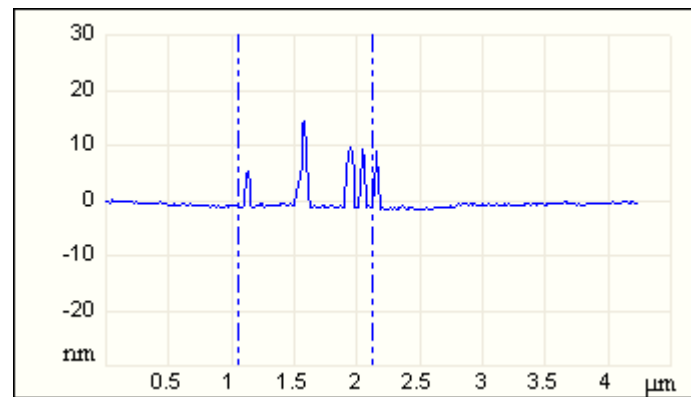

Supplement: S2 Fig — (PDF) [file pone.0136602.s002.pdf]
